# Transcatheter Arterial Embolization for Blunt Trauma in a Horseshoe Kidney With Contralateral Renal Artery Anomaly: A Case Report and Literature Review

**DOI:** 10.7759/cureus.78614

**Published:** 2025-02-06

**Authors:** Miho Yoshimatsu, Hiroyuki Tokue, Akiko Jingu, Takakazu Komatsu, Yoshito Tsushima

**Affiliations:** 1 Department of Diagnostic Radiology and Nuclear Medicine, Gunma University Hospital, Maebashi, JPN

**Keywords:** active arterial bleeding, horseshoe kidney, renal injury, transcatheter arterial embolization, vascular anomaly

## Abstract

Horseshoe kidney (HSK) is a common renal anomaly associated with an increased risk of traumatic injury due to its superficial location and frequent vascular anomalies. We report a case of a 23-year-old male patient with HSK and a rare vascular anomaly, a right renal artery originating from the left common iliac artery, who sustained a grade V renal injury following blunt trauma. Contrast-enhanced computed tomography (CT) revealed active arterial bleeding and a retroperitoneal hematoma. Emergency transcatheter arterial embolization (TAE) successfully controlled active bleeding. The patient recovered without complications. This case underscores the importance of pre-procedural vascular evaluation in HSKs, as well as the efficacy of TAE in managing severe renal injuries associated with complex vascular anatomy.

## Introduction

Renal injuries occur in 10% of blunt abdominal traumas, with 7% of these occurring in kidneys with congenital or acquired disorders, such as horseshoe kidney (HSK) [1]. HSK is a common renal anomaly occurring in approximately 0.25% of the population [2], an anomaly characterized by the fusion of the lower poles of the kidneys and an isthmus located in the midline [3]. Owing to its superficial location and lack of protection by the thoracic cage, an HSK is considered vulnerable to traumatic injury [3]. Management of kidney injury in HSK depends on the severity of the trauma. Conservative management with close monitoring is preferred in hemodynamically stable patients, while interventional radiology techniques, such as angioembolization, may be required in cases of active bleeding [4]. In severe injuries with hemodynamic instability, surgical intervention, including partial or total nephrectomy, may be necessary [4]. Other treatment options include repair of the renal artery or renal vein, but failure rates are high in cases of severe renal injury [4]. However, given the frequent vascular anomalies associated with HSK [2], pre-procedural identification of vascular anatomy is crucial.

Here, we report a case of blunt trauma in a patient with HSK who exhibited a rare vascular anomaly where the right renal artery originated from the left common iliac artery. The patient was successfully treated with transcatheter arterial embolization (TAE). This report highlights the challenges associated with diagnosing and managing renal trauma in a patient with HSK, emphasizing the need for emergency physicians and surgeons to maintain a high index of suspicion in such cases. Given the anatomical variations in HSK, early imaging and a multidisciplinary approach are crucial for optimal patient outcomes.

## Case presentation

The patient was a 23-year-old man with an unremarkable medical history or medication. He was involved in a single-vehicle collision with a concrete barrier while driving at 40-50 km/hour. He presented with right lower abdominal pain and was transported to our emergency department.

On arrival, the patient was alert with stable vital signs: blood pressure of 113/84 mmHg, pulse rate of 60/minute, and SpO₂ of 99% on room air. However, he appeared distressed, and his blood pressure temporarily fell to 71/52 mmHg. He vomited multiple times and exhibited tenderness and rigidity in the right flank. Seatbelt marks were noted on his right shoulder and flank. Focused assessment with sonography for trauma was negative, and hematuria was not observed. Laboratory findings revealed no renal dysfunction (creatinine: 0.9 mg/dL, reference range: 0.6-1.3 mg/dL; estimated glomerular filtration rate (eGFR): 80.6 mL/minute/1.73 m², reference range: ≥90 mL/minute/1.73 m²); however, there was mild anemia (hemoglobin: 11.7 g/dL), prompting transfusion of two units of red blood cells.

**Figure 1:**
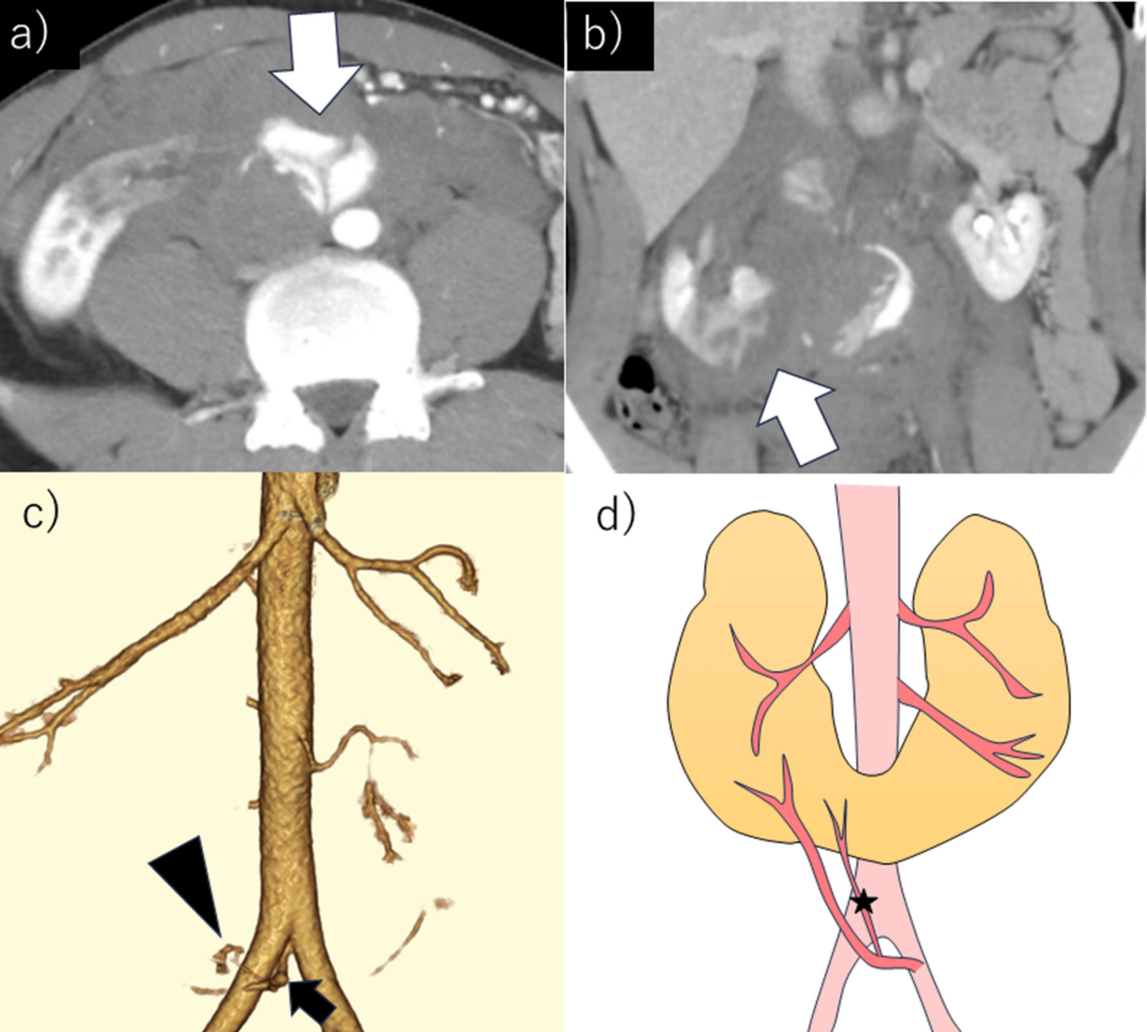
The patient was a 23-year-old man who presented with right lower abdominal pain following a self-inflicted car accident. Contrast-enhanced CT was performed. (a) Axial view: Perirenal hematoma and early enhancement (arrow) are observed. (b) Coronal view: Arterial bleeding at the renal isthmus and poor contrast enhancement at the lower pole of the right kidney (arrow) are noted. (c) 3D-CT: Active bleeding (arrowhead) originated from a branch of the right renal artery at the lower pole (arrow), which branched from the left common iliac artery. (d) Schematic representation of the 3D-CT findings: A single right renal artery and two left renal arteries originated from the aorta, with an additional branch of the right renal artery at the lower pole branching from the left common iliac artery. Active bleeding (star) continuous with the branch was identified. CT: computed tomography, 3D-CT: three-dimensional computed tomography

Contrast-enhanced computed tomography (CT) revealed active arterial bleeding associated with a ruptured HSK, retroperitoneal hematoma, and decreased perfusion of the renal isthmus (Figure 1a, 1b). The renal injury was classified as grade V according to the American Association for the Surgery of Trauma classification [2]. Active bleeding originated from a branch of the right renal artery, which was found to arise from the left common iliac artery (Figure 1c, 1d).

**Figure 2:**
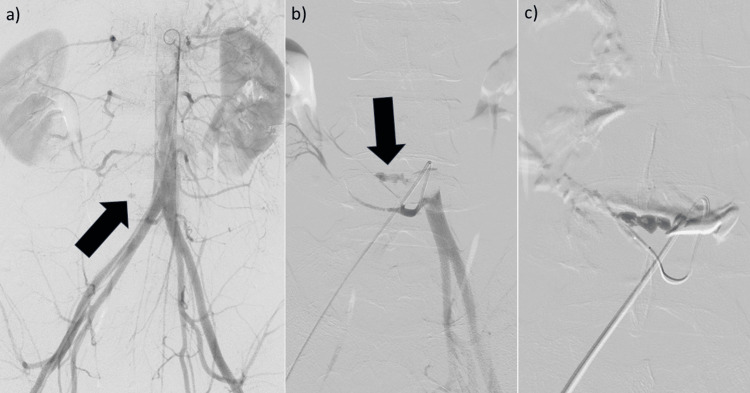
Emergency transcatheter arterial embolization was planned for active arterial bleeding associated with a ruptured HSK. (a) A 5-Fr-long sheath was placed in the right femoral artery. Aortography using a 4.2-Fr pigtail catheter shows vascular extravasation (arrow) on the right side of the aorta. (b) Selective angiography of the left common iliac artery using a 5-Fr Shepherd hook catheter reveals extravasation from the branch of the right renal artery at the lower pole (arrow). (c) A 1.9-Fr microcatheter was advanced to the bleeding site, and embolization was performed using N-butyl cyanoacrylate and lipiodol (1:3) along with gelatin sponge particles. HSK: horseshoe kidney

Emergency TAE was planned. A 5-Fr-long sheath was inserted via the right femoral artery, and a 5-Fr shepherd hook catheter was used to select the aberrant right renal artery branch arising from the left common iliac artery. Angiography confirmed active extravasation (Figure 2a, 2b). A 1.9-Fr microcatheter was advanced to the bleeding site, and embolization was performed using a mixture (2cc) of N-butyl cyanoacrylate (NBCA) and lipiodol (1:3) along with gelatin sponge particles (Figure 2c). Hemostasis was successfully achieved.

**Figure 3:**
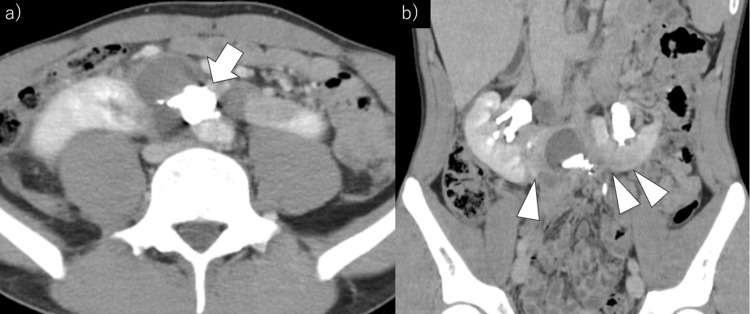
Contrast-enhanced computed tomography was performed one month after the procedure. (a) Axial view: Good deposition of N-butyl-2-cyanoacrylate and lipiodol (arrow) and reduction of the retroperitoneal hematoma are observed. (b) Coronal view: Poor contrast enhancement in the renal isthmus and both renal lower poles (arrowheads) is observed, but no extension of the renal infarction is noted.

Post-procedural recovery was uneventful, with no rebleeding or hemodynamic instability. On day 5 of the hospital stay, follow-up non-contrast CT demonstrated reduced retroperitoneal hematoma and deposition of lipiodol within the hematoma. The patient resumed eating on day 4 and walking on day 12 and was discharged in good general condition on day 17. During hospitalization, renal function remained stable (eGFR: 75-85 mL/minute/1.73 m²). At one month of follow-up, contrast-enhanced CT revealed ischemic infarction of the renal isthmus without further progression, and the retroperitoneal hematoma had further reduced in size (Figure 3a, 3b).

**Figure 4:**
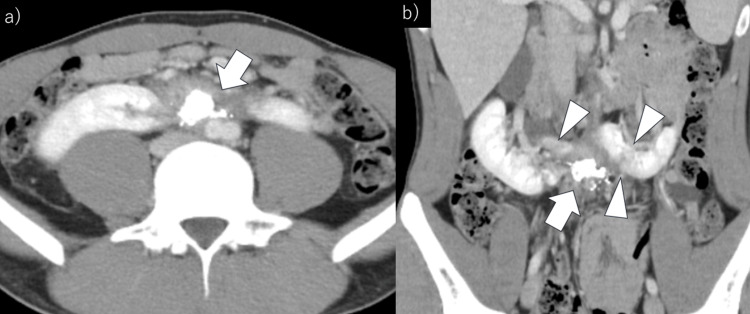
Three months after the procedure, follow-up contrast-enhanced computed tomography was performed. (a) Axial view: Further reduction of the retroperitoneal hematoma (arrow) is observed. (b) Coronal view: Retroperitoneal hematoma (arrow) is reduced, and the area of renal isthmus infarction (arrowheads) is decreased.

At three months, the infarction area had diminished, and the hematoma showed further resolution (Figure 4a, 4b).

## Discussion

An HSK is a common renal anomaly occurring in approximately 0.25% of the population, with various renal artery anomalies having been well-documented [2]. One of the earliest classifications proposed by Eisendrath et al. in 1925 categorized renal artery patterns in HSKs into five types [5]: Type I, with a single pair of renal arteries on both sides; Type II, with an additional duplicated renal artery arising from the aorta; Type III, with two pairs of renal arteries on both sides and duplicated arteries supplying the isthmus; Type IV, with duplicated renal arteries from the aorta and one or more arteries from the iliac arteries to the isthmus; and Type V, with multiple duplicated arteries originating from the aorta, mesenteric arteries, and iliac arteries. Reported frequencies are as follows: Type I, 20%; Type II, 30%; Type III, 15%; Type IV, 15%; and Type V, 20% [6].

In 1988, Crawford et al. introduced a classification based on the number of arterial origins, categorizing HSK vascular patterns into three types [7]: Type I, with a single pair of renal arteries originating from the normal location; Type II, with a single pair of renal arteries and 1-4 ectopic arteries branching from the aorta or iliac arteries; and Type III, with multiple small arteries arising from the aorta or iliac arteries. This classification is considered particularly useful for surgical planning [8].

Recognizing that no single classification could encompass all cases, Hekimoglu et al. proposed a new classification in 2023 [9]. This system defines five types: Type 1, where accessory arteries are absent or originate bilaterally from the abdominal aorta; Type 2, which includes accessory arteries from the midline abdominal aorta or sacral arteries; Type 3, with accessory arteries originating from the common iliac artery; Type 4, involving accessory arteries from both the midline aorta or sacral arteries and common iliac artery; and Type 5, where accessory arteries originate from the superior mesenteric artery. The reported frequencies are as follows: Type 1, 13.1%; Type 2, 57.2%; Type 3, 17.2%; Type 4, 10.3%; and Type 5, 1.4%.

In the present case, the patient exhibited a single right renal artery and two left renal arteries branching from the aorta, as well as a right renal artery originating from the left common iliac artery. None of the classifications by Eisendrath et al., Crawford et al., or Hekimoglu et al. explicitly accounted for renal arteries originating contralaterally from the iliac artery. To the best of our knowledge, no prior reports have described contralateral iliac artery origin in HSKs, although three cases have been reported in normal kidneys, all involving the right renal artery originating from the left common iliac artery [10-12].

Krutsri et al. reviewed eight cases of blunt trauma in HSKs, with a mean patient age of 31.75 years and a predominance of male patients [13]. Hematuria and retroperitoneal hemorrhage were common clinical presentations, with active bleeding detected on CT in 62.5% (5/8) of cases. Of these, four underwent endovascular treatments such as TAE or stenting [13].

Treatment options for renal injury include vascular embolization and surgical intervention. Surgical approaches consist of partial nephrectomy and total nephrectomy. In some cases, repair of the renal artery or renal vein may be performed; however, in cases of grade V renal injury, such as the present case, the failure rate of vascular repair is high, and nephrectomy is often the preferred treatment [4].

Owing to the high incidence of vascular anomalies in HSKs, nephron-sparing surgery can be challenging, making endovascular treatments, such as TAE, a preferable option [13]. Recently, TAE for even severe renal injury has been used [4]. The current case involved a grade V renal injury with active bleeding, making TAE the treatment of choice.

A PubMed search using the keywords "horseshoe kidney," "trauma," and "embolization" identified six cases of blunt trauma in HSKs treated with TAE (Table 1) [13-18]. The mean patient age was 41.5 years, and all patients were males. Trauma causes included motor vehicle accidents (n=2), soccer injuries (n=1), falls (n=1), self-inflicted stab wounds (n=1), and unspecified abdominal trauma (n=1). The vessels responsible for bleeding were branches of the lower pole of the right renal artery (n=3), lower pole of the left renal artery (n=1), upper pole of the right renal artery (n=1), and an accessory artery originating from both the left renal and left common iliac arteries (n=1). According to the Hekimoglu classification, there were two Type 1 cases, one Type 3 case, and three unclassified cases. Embolization materials included coils (n=5) and non-absorbable agents (n=2). All the cases achieved successful embolization, with three reporting early discharge (2-5 days post-procedure). Two cases had complications: one developed retroperitoneal fluid collection treated with percutaneous drainage, and the other experienced an infected hematoma managed with ceftriaxone.

**Table 1:** Literature review of blunt trauma with horseshoe kidney treated with TAE. All cases involved male patients, and the TAE procedures were successful in all cases. NBCA-LPD: N-butyl-2-cyanoacrylate and lipiodol, TAE: transcatheter arterial embolization

Author	Age	Origin of the trauma	Responsible artery of the bleeding	Hekimoglu's classification	Embolic materials	Complications
Legg et al. [14]	49	Falling from 15 feet	Left lower pole accessory renal artery	No data	2 coils (2 mm)	A large retroperitoneal fluid collection (two months later) →a 10-Fr drainage catheter percutaneously
Molina Escudero et al. [15]	25	Playing football	Lower branch of the right renal artery	1	Non-absorbable material	None
Anil et al. [16]	21	Motorbike accident	Upper fragment of the right renal artery	No data	Coil (4 × 14 mm), gel foam	An infected hematoma (7 days later) →ceftriaxone without surgery
Pezeshki Rad et al. [17]	19	Self-stab wound	Segmental branch of the right lower pole	1	Coil (3 mm)	None
Krutsri et al. [13]	57	Motorcycle road traffic accident	Lower branch of the right renal artery	No data	Coil	None
Boninsegna et al. [18]	78	Abdominal trauma	(1) Left renal artery, (2) accessory renal artery from the left common iliac artery	3	(1) 2 coils (2 × 25 mm and 2 × 50 mm), (2) 3 coils (2 × 25 mm, 2 × 25 mm, and 3 × 30 mm)	None
Our case	23	Self-inflicted car accident	Lower branch of the right renal artery from the left common iliac artery	Unclassifiable	NBCA-LPD (1:3), gelatin sponge	None

In the present case, successful embolization was achieved, with subsequent resolution of the hematoma confirmed.

## Conclusions

HSK trauma cases requiring TAE have been rarely reported. However, previous reports indicate that complications are uncommon, and in this case as well, no TAE-related complications were observed, demonstrating its effectiveness as a treatment option. Given the high incidence of vascular anomalies in HSKs, including rare cases like the present one where the renal artery originates from the contralateral common iliac artery, a thorough understanding of the vascular anatomy is crucial when performing TAE. Moreover, emergency physicians and surgeons should maintain a high level of vigilance in such cases, as early recognition of this anatomical variation is essential for selecting an appropriate treatment strategy, particularly in cases of active bleeding.
